# A prospective study assessing feasibility of performing percutaneous nephrolithotomy in chronic kidney disease patients - What factors affect the outcome?

**DOI:** 10.1590/S1677-5538.IBJU.2018.0816

**Published:** 2019-09-02

**Authors:** Rohan Patel, Samarth Agarwal, S. N. Sankhwar, Apul Goel, B. P. Singh, Manoj Kumar

**Affiliations:** 1 Department of Urology, King George’s Medical University, Lucknow, Indiaw

**Keywords:** Nephrolithotomy, Percutaneous, Kidney Diseases, Urinary Tract Infections

## Abstract

**Objectives:**

To primarily evaluate the functional outcomes of PCNL for bilateral renal calculi/calculi in solitary functioning kidney with Chronic Kidney Disease(CKD). To identify factors affecting the renal replacement therapy following PCNL.

**Materials and Methods:**

Patients with bilateral renal calculi/calculi in solitary kidney and CKD (eGFR<60/s.creatinine>2) and Good Performance Status [Eastern Cooperative Oncology Group (ECOG): 0–2] were included in the study.

**Results:**

A total of 60 patients with CKD who had bilateral renal calculi/calculi in solitary functioning kidney underwent PCNL. At 6 months, eGFR improved or stabilized in 45 (75%) patients, while in 15 (25%) patients eGFR deteriorated. A total of 5 (14.28%) and 2 (25%) patients of CKD stage 4 and 5 respectively had improvement in eGFR as well as CKD stage. Fourteen (82.35%), 21 (60%), 3 (37.5%) patients of CKD stage 3, 4, 5 had improvement in eGFR but not significant enough to cause stage migration. Again 3 (17.65%) , 9 ( 40%) and 3 (37.5%) patients of CKD stage 3, 4, 5 had reduction in eGFR but not significant enough to cause stage migration. None of the patients had worsening of CKD stage. Preoperative CKD stage and eGFR were compared with measurements made at the final follow up visit (6 months).

**Conclusion:**

Our results indicate that most patients of renal calculi with CKD show improvement or stabilization of renal function with aggressive stone removal. Improvement is more in patients who have mild to moderate CKD. Aggressive management of comorbidities, peri-operative UTI and complications may delay or avoid progression of CKD status in such patients.

## INTRODUCTION

Chronic kidney disease(CKD) is a common public health disorder that is defined as sustained kidney injury of more than 3 months resulting in a GFR of less than 60 mL/ min/1.73 m^2^ by Kidney Disease Outcomes Quality Initiative (K/DOQI) Advisory Board (1). Current estimates suggest that chronic kidney disease affects 10%-13% of the adult American population (2). In India, it has been recently estimated that the age-adjusted incidence rate of ESRD is 229/million population, and >100,000 new patients enter renal replacement programs annually (3).

Patient with CKD represent 0.8%-17.5% of those presenting with urinary stone disease (4, 5). The incidence of developing end stage renal disease (ESRD) in patients with renal calculi is 0.2-3.2% (6). The aetiology of renal insufficiency in patients with nephrolithiasis is multifactorial and includes renal obstruction, recurrent urinary tract infections, frequent surgical interventions and coexisting medical disease (4, 5, 7).

Patients with chronic kidney disease frequently have various medical comorbidities, such as diabetes, hypertension, anaemia and bleeding disorders. The likelihood of comorbid conditions may increase the operative risk, the incidence of postoperative complications, and negatively impact the success rate.

Gupta et al. reported that 75.8% of patients with urinary stone disease and deranged renal function requires multiple procedures for stone clearance, including ESWL, PCNL, uretero-renoscopy and open surgical procedures (4).

In the modern era, percutaneous nephrolithotomy (PCNL) has emerged as the gold standard intervention for large burden and complex renal stone disease and is associated with the highest stone free rates (SFRs), usually in a single setting. However, potentially significant complications include bleeding (requiring blood transfusion or embolization), sepsis, pleural and visceral injury. Therefore, the optimal management plan needs to be tailored to individual patient (8).

Many studies have shown the long-term safety of PCNL in patients with normal renal function. However, there are limited data on the outcomes of patients with CKD who undergo PCNL. In this study, we prospectively evaluated the outcomes of renal function in patients with bilateral renal calculi/calculi in solitary kidney with CKD who underwent PCNL, and determined the factors affecting outcome.

## AIM AND OBJECTIVES

Primary end point - To evaluate the functional outcomes of PCNL for bilateral renal calculi/calculi in solitary functioning kidney with CKD.

Secondary end point - To identify factors affecting the renal replacement therapy following PCNL in CKD patients.

## MATERIALS AND METHODOLOGY

### Place of Study

This prospective study was conducted at the Department of Urology, King George’s Medical University, Lucknow from October 2015 to October 2017 after Institutional review board clearance.

### Consent

Written informed consent was taken from all the patients included in this study.

### Inclusion Criteria

1. Patients with bilateral renal calculi/calculi in solitary kidney and CKD (eGFR<60/s.creatinine>2)
2. Good Performance Status [Eastern Cooperative Oncology Group (ECOG): 0 – 2]

### Exclusion Criteria

1. Poor performance status [Eastern Cooperative Oncology Group (ECOG) >2]
2. Patient not giving consent for PCNL
3. Uncorrected bleeding diathesis
4. Pregnancy

## MATERIALS AND METHODS

Initial clinical data including complete blood count, random blood sugar (RBS), serum urea and serum creatinine, electrolytes (sodium, potassium, calcium), prothrombin time (PT and INR), urine routine and culture sensitivity(c/s), X-ray KUB, ultrasound KUB and non-contrast computerized tomography (NCCT) KUB were recorded. Stone complexity was calculated using Guy’s stone score (9). The eGFR for each patient was calculated using a 4-variable MDRD equation (10). CKD was classified using the National Kidney Foundation Kidney Disease Outcome Quality Initiative classification system (11). Pre PCNL serum creatinine and eGFR measurement were done 1 day before the surgery.

Performance status was evaluated using Eastern Cooperative Oncology Group (ECOG) Scale (12).

Renal decompression with either Double-J (DJ) stent or percutaneous nephrostomy(PCN) was done for obstructed and infected units. Post-decompression renal function was assessed serially and patients underwent surgery only after stabilization of eGFR reading recorded at two separate settings.

Antibiotic therapy was given to all patients who had positive urine cultures till documentation of sterile urine. Nephrology consultation was taken regarding optimization of comorbidities and perioperative renal replacement therapy. Appropriate Renal Replacement therapy was given whenever required and as advised by the nephrologist. All PCNL’s were performed at our center by senior consultant urologists or resident trainees under faculty supervision.

## SURGICAL TECHNIQUE

Following induction of anesthesia, a 5-Fr ureteral catheter (open-ended) was placed to the stone side in lithotomy position via cystoscopy. After returning to prone position, the anatomy of collecting system was delineated using a radiocontrast medium and/or air under fluoroscopy guidance. Puncture and dilatation of the tract was done as per Bull’s eye technique using Amplatz dilators, and procedure performed with a 24-French rigid nephroscope (Richard Wolf, Germany), pneumatic lithotripter (swiss lithoclast®), and grasping forceps. All fragments that were accessible by a rigid nephroscope were removed with a grasping forceps. At the end of procedure, a 20-French nephrostomy tube and 5 French DJ stent were placed in all patients as per our institutional protocol.

Operative time was defined as time elapsed from induction of anesthesia till termination of procedure. Site of puncture (supra vs infra-costal), number of punctures, size of tract, and number of sessions were recorded for each patient.

On postoperative day 1, X-ray KUB and/or USG KUB (for radiolucent calculi) was done in all patients to document stone clearance. The decision of removing the nephrostomy tube was based on nephrostomy tube output, normal intra-operative nephrostogram and normal postoperative X-ray KUB/renal ultrasound (in case of radiolucent calculi). Patients were discharged next morning after removal of per-urethral catheter.

Complete stone clearance was defined as no visible calculi in X ray or NCCT KUB. Clinically insignificant residual fragments were defined as <4 mm. These patients were managed conservatively according to European Association of Urology guidelines (13).

Complications were recorded according to modified Clavien-Dindo classification of postoperative complications (14). In patients with bilateral calculi, PCNL was performed on 2^nd^ side after an interval of 1 month.

All patients were followed up at 2 weeks (for DJ stent removal) and at 6 months when urinalysis, serum creatinine, X-ray KUB and USG KUB, serum creatinine and eGFR were measured. Preoperative CKD stage and eGFR were compared with measurements made at 6 months follow-up visit. Patients were divided into 2 groups by changes in CKD (eGFR) status:

Group 1- improved or stable disease and

Group 2- worsened disease.

The effects of independent variables on kidney function after PCNL, were evaluated by comparing the two groups.

### Statistical considerations

The data were entered in an Excel database and analysed with an SPSS version 21.0 (IBM SPSS statistics 21 SPSS Inc.) statistical software package using the Chi-square test, Student’s t-test, and Fischer exact test. P < 0.05 was considered as statistically significant.

## OBSERVATION AND RESULTS

A total of 60 patients with CKD who had bilateral renal calculi/calculi in solitary functioning kidney underwent PCNL during the period from October 2015 to October 2017. Thirty-one patients had bilateral renal stones while the remaining 29 patients had calculi in solitary functioning kidney (congenital absent kidney-3, history of nephrectomy-2, congenital atrophic kidney-24). Thus, a total of 91 renal units in 60 patients underwent PCNL.

## PREOPERATIVE PARAMETERS

### Demographic parameters

Mean ± SD age was 43.16 ± 16.3 years, the youngest being 12 and the eldest being 75 years of age. 43 (71.67%) were male and 17(28.33%) were female. 12 (20%) patients had history of previous open surgery while 48 (80%) did not. None had a history of PCNL or ESWL. Fifty two patients had presented with anuria and underwent some form of urinary diversion either with DJ stenting or percutaneous nephrostomy.

Patients were preoperatively classified as having CKD stage 1, 2, 3, 4, 5 respectively according to KDOQI classification. None had stage 1 or 2 CKD while 17, 35 and 8 patients were classified as CKD stage 3, 4 and 5 respectively. Stone complexity was given by Guys stone score and 30, 23, 23, 15 renal units had Guys stone score of 1, 2, 3, 4 respectively.

## OPERATIVE PARAMETERS:

### Operative duration:

Mean ± SD operative time was 120.01 ± 38.24 minutes/renal unit. Operative duration was >100 mins in 62 renal units. A single puncture was used in 44 renal units, while 47 renal units required multiple punctures. Amongst these 25 (27%) renal units required supracostal punctures, while in 66 (73%) kidneys infracostal puncture was achieved.

## COMPLICATIONS

A total of 46 complications were noted in 20 patients which was summarized according to Clavien Dindo classification. Bleeding necessitating transfusion (26.6%) was the most common complication. Seven (11.6%) patients developed fever that resolved with antipyretics. Ten (16.67%) patients developed UTI necessitating antibiotic therapy. Three (5%) patients developed seizures in immediate postoperative period, that were managed by anticonvulsants. Three (5%) patients developed urosepsis necessitating antibiotics, vasopressors and fluid resuscitation. DJ replacement for prolonged urine leak was required in 4 (6.67%) patients, 3 of whom had urinoma. No mortality was seen in our study. There were no Grade 3b, 4a, 5 complications.

Stone clearance was complete in 49 patients (81.6%) (defined as no residual calculi on NCCT KUB or X-ray KUB) after PCNL.

As an auxiliary treatment, ESWL was done in 2 patients, ureteroscopy in 4, while 5 patients with asymptomatic clinically insignificant residual stones (<4 mm) were followed conservatively. Spontaneous stone passage was seen in 2 patients who were followed conservatively at 3 months.

## FOLLOW UP

### eGFR value during follow-up

At 6 months follow up, eGFR improved or stabilized in 45 (75%) patients, while in 15 (25%) patients eGFR deteriorated.

### CKD stage at follow-up

At 6 months follow up, a total of 5 (14.28%) and 2 (25%) patients of CKD stage 4 and 5 respectively had improvement in eGFR as well as CKD stage. Fourteen (82.35%), 21 (60%), 3 (37.5%) patients of CKD stage 3, 4, 5 had improvement in eGFR but not significant enough to cause stage migration. Again 3 (17.65%), 9 ( 40%) and 3 (37.5%) patients of CKD stage 3, 4, 5 had reduction in eGFR but not significant enough to cause stage migration. None of the patients had worsening of CKD stage.

**Figure 1:**
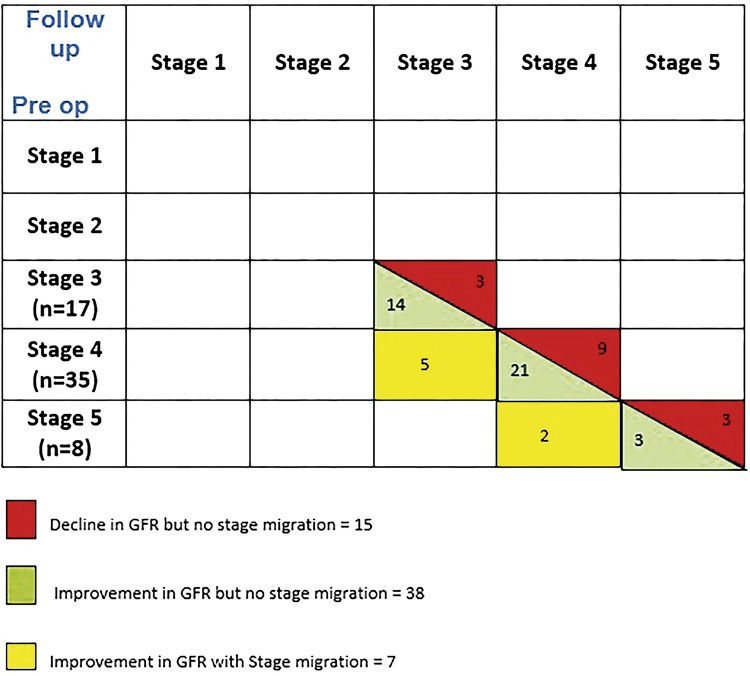
Comparison of pre op and post op GFR and stage migration.

These changes are shown in Figure-1.

Preoperative CKD stage and eGFR were compared with measurements made at the final follow-up visit (6 months). Patients were divided into 2 groups by changes in CKD stage, including:

Group 1-improved or stable disease and Group 2-worsened disease since the final follow-up visit.

The effects of independent variables on kidney function after PCNL, including patients age, gender, history of open surgery, comorbid diseases (DM and HTN), stone complexity (GSS), hydronephrosis degree, number of access sites, operative duration, peri-operatory complications, stone-free status at postoperative month 6 and recurrent urinary infections during follow up, were evaluated by comparing the 2 groups as shown in Table-1.

**Table 1:** Univariate analysis of patient and procedure related factors affecting kidney function after PCNL.

Variable	Group 1(n)	Group 2 (n)	P value
**eGFR (Mean) mL/min/1.73m**^**2**^			
Baseline	25.65	22.44	0.20
Follow-up	29.07	20.71	**0.004**
Age(Mean ± SD)years	41.6 ± 15.5	47.13 ± 18.2	0.25
**Gender**			**0.61**
M	33	10	
F	12	5	
Diabetes	3	4	**0.03**
Hypertension	7	5	0.13
Previous open surgery	7	5	0.13
**Hydronephrosis grade**			**0.27**
Mild to moderate	60	17	
Severe	9	5	
**Guys Stone Score**			**0.92**
1-2	40	13	
3-4	29	9	
**CKD stage**			**0.55**
1	0	0	
2	0	0	
3	14	3	
4	26	9	
5	5	3	
**Access no.**			**0.85**
Single	33	11	
Multiple	36	11	
Supracostal puncture	20	5	0.56
**Operative time**			**0.59**
<100 min.	23	6	
>100 min.	46	16	
**Blood transfusion due to bleeding**			**0.72**
Yes	10	4	
No	35	11	
**Stone free status**			**0.08**
Yes	39	10	
No	6	5	
**Complications**			**0.01**
Yes	11	9	
No	34	6	
**Recurrent UTI**			**0.03**
Yes	3	4	
No	42	11	

On univariate analysis, diabetes, peri-operatory complications, patients with history of recurrent UTI and eGFR at follow up were found to be the significant factors affecting outcome.

A multivariable analysis using a logistic regression model was used to determine if any of the potential risk factors was also associated with risk of renal replacement in future. The independent risk factors identified as predictors of RRT were eGFR (coefficient 2.85, P = 0.025), degree of hydronephrosis (coefficient 2.10, P = 0.04) diabetes ( coefficient 1.67, P = 0.045) and recurrent UTI ( coefficient 2.50 P= 0.034).

## DISCUSSION

CKD is a major public health problem, and in the surgical setting, not only is it associated with higher risk of anesthetic complications, but also greater risk of post-procedure complications (15). In addition to achieving good stone clearance, surgical interventions employed in the treatment of stone disease must try and preserve maximal renal function. Management of nephrolithiasis in patients with CKD is therefore a difficult challenge for the endourologist as well as nephrologists and calls for careful consideration of the risks against the benefits.

In our study, mean age of patients was 43.16 ± 16.3 years which was slightly lower compared to studies by Kurien et al. (16), Bilen et al. (17), Kumar et al. (18), Akdeniz et al. (19) where the reported mean age of distribution varied from 45-59.5 years. This could be due to larger number of patients of younger age group (<20 years, 6 patients) in our study compared to these studies.

The mean preoperative eGFR was 24.9 ± 8.56 (mL/min/1.73 m^2^), which was lower than that reported in literature (15-17, 19, 20). This could be due to the inclusion criteria of higher serum creatinine > 2 mg/dL in our study, which was higher than inclusion criteria (eGFR <60/Serum creatinine >1.5) taken in these studies.

Seven (11.6%) patients had diabetes. This was comparable to the incidence of DM in studies reported by Akdeniz et al. (19), Akman et al. (15), Sairam et al. (21) and 12 (20%) patients had hypertension. There has been large variation in the reported incidence (8.6-42%) of hypertension in other studies (15, 17, 19). Jones et al. (8) in a systemic review of 9 studies (n=1851), reported 30.7% incidence rate of hypertension which was comparable to our study.

The mean operative time was 120.01 ± 38.24 (range 60-250) minutes, which was higher than those reported in literature (15, 18, 19). This could be due to trainees performing some of the procedures, leading to longer operative time or due to more complex stones (43% GSS 3 or 4). None of other studies reported stone burden in form of GSS but mean stone size in these studies was 706.8 mm2 (range 357-1484 mm^2^).

Single access was gained in 48.3% of cases with a mean of 1.57 punctures/renal unit. This was lower compared to various studies that report frequency of single access to be 68-80%. This could be due to the high frequency of complex calculi (GSS 3-4, 41.7%) which required multiple access to achieve maximal stone clearance. Complete stone clearance was achieved in 81.6% cases which is also in concordance with that reported in literature (70-90%) (15-19).

### Complications

Seven (11.6%) patients developed fever (grade 1) which was managed successfully by conservative management. Blood transfusion due to postoperative drop in haemoglobin was the most common complication seen in 16 (26.6%) patients. This was similar to the reported incidence of blood transfusion in various studies from 9.6-36% (16-19). Such high rates of blood transfusion could be attributed to pre-existing anemia and platelet dysfunction in CKD patients. None of the patients reported with delayed haemorrhage or required angioembolization.

Seizures was seen in 3 patients in immediate postoperative period which was managed successfully by anti-convulsants. Seizures could be attributed to altered anaesthetic drug metabolism or electrolyte imbalance seen in CKD patients.

DJ stent was routinely done in all our patients. However, 9 patients developed urinary leakage following nephrostomy removal. In five patients it subsided by conservative management like anticholinergics and compression dressing. In 4 patients who did not respond to conservative management, DJ stent replacement was done following which it resolved. Akman et al. (15) in his series of 177 patients reported 2.2% incidence of DJ stenting for prolonged urine leak. Ansari et al. (22) in their study of 576 patients, found stone complexity, grade of hydronephrosis, renal parenchymal thickness in access line, intra-parenchymal renal pelvis, multiple punctures, surgeon’s experience, and residual stones as factors for prolonged urinary leakage post-PCNL. Possible explanation of this high incidence (6.6%) of prolonged urine leak in our study could be multiple punctures (2 patients), gross hydronephrosis (1 patient) and presence of CKD which is associated with delayed wound healing.

Urosepsis (Grade 4b) occurred in 3 (5%) patients, which was similar to incidence reported in literature of 2.8-9.9% (15-17). Such high incidence of sepsis could be due to immune-deficiency in CKD patients, staghorn calculi, presence of DJ stent/PCN in patients. All of these patients were managed aggressively in ICU setting with antibiotics, fluid resuscitation, vasopressors. No mortality was seen.

## FOLLOW-UP RENAL FUNCTION

Mean eGFR during the preoperative period, and at 6 months follow-up was 24.9 ± 8.56 and 27 ± 10.13 mL per minute/1.73 m^2^ respectively. Overall renal function improvement was seen in 45 (75%) patients, while in 15 (25%) patients it deteriorated. This was in concordance with overall improvement in renal function seen in reported literature (15-19). Fourteen (82.3%), 26 (64.28%) and 5 (62.5%) patients in stage 3, 4, 5 showed improvement in eGFR post intervention respectively. Kurien et al. in their study suggested that an improvement in eGFR was greater in patients with mild to moderate renal failure than in those with severe CKD (16). It would be reasonable to assume that those patients with severe renal failure would be less likely to gain benefit, principally because the damage already done to the kidney was severe and irreversible.

However, in a study done by Bilen et al., patients with late stage CKD, although a small number, achieved significant improvement, while unexpected deterioration was seen in some patients with less severe CKD (17).

Urinary tract infection appeared to be the underlying cause of the observation in the latter study, emphasizing the need for constant vigilance against infection in all PCNL cases regardless of CKD status. However studies agreed that through ever more aggressive stone removal and more effective prevention of infection, renal replacement therapy can still be deferred in most patients with renal stone disease (16, 17).

The results of other studies are summed up in Table-2.

**Table 2:** Different studies comparing various preop, intra op and post op characteristics.

Study	Age Mean (SD, range) years	Diabetes	Inclusion criteria (eGFR (mL/min./1.73 m^2^)	Mean Pre-Op. eGFR (mL/min./1.73 m^2^)	Mean Operative time Min. (SD,range)	Complication Haemmorhage requiring		Follow up eGFR(mL/min/1.73m^2^)	Overall change

						Transfusion	Sepsis		
**Present study**	43.16 (16.38, 12-75)	11.6%	<60/S. Creatinine >2	24.9 ± 8.56	120.01 (38.24, 60-250)	26.6%	5%	27	improved
Bilen et al. (17)	53.2 (NR, 20-81)	1.62%	<60	S. Creatinine 1.87	101.4 (NR, NR)	36%	3%	48.4	improved
Kurien et al. (16)	52.5 (13, NR)	-	S. Creatinine >1.5	S. Creatinine 3.2	NR	20.5%	9.9%	43.3	improved
Akman et al. (15)	54.3 ± 12.1 years	19.8%	<60	44.8	65.12 (22.83, NR)	9.6%	2.8%	48	improved
Ozden et al. (20)	-	-	<60	39.9	NR	-	-	51.3	improved
Akdeniz et al. (19)	59.5 (7.85, 39-78)	11.8%	<60	44.8	NR	11.8%	NR	51.8	improved
Sairam et al. (21)	-	18.8%	-	-	89 (52.3, NR)	-	-	-	improved
Kumar et al. (18)	45 (NR, 18-65)	-	s.creat. >4.5	s.creat. -6.3	110 (32,NR)	20%	20%	2.56 (s.creatinine)	improved

On univariate analysis, diabetes (p=0.03), perioperative complications (p=0.01) and recurrent UTI (p=0.03) were found to be significant factors that negatively impact the outcome.

Akman et al. (15) in their study of long-term outcomes of percutaneous nephrolithotomy in 177 patients with chronic kidney disease, found that diabetes and preoperative or postoperative complications predicted renal function on multivariate regression analysis. Kurien et al. (16) in their study of 91 patients found postoperative complications and peak eGFR (less than 30 mL/minute/1.73 m^2^) at follow-up to predict renal deterioration and need for renal replacement therapy(RRT). Renal parenchymal thickness (<8 mm) also predicted the need for RRT.

Bilen et al. (17) in their study found that only the presence of urinary-tract infections had a tendency to negatively affect the GFR.

Ozden et al. (20) reported that diabetes mellitus (odds ratio 15.82, P=0 .036) and urinary infection (odds ratio 10.6, P=0.04) were predictive of renal function deterioration at 1 year on multivariate analysis.

Our results indicate that most patients of renal calculi with CKD show improvement or stabilization of renal function with aggressive stone removal. Improvement is more frequent in patients who have mild to moderate CKD. Although complications are higher in CKD patients most are of low grade, thereby confirming the safety and efficacy of PCNL in CKD patients. Aggressive management of comorbidities and perioperative UTI and complications in these patients may delay or avoid progression of CKD status in these patients.

## LIMITATIONS OF OUR STUDY

Our study comprised of only 60 patients with limited follow-up period of 6 months. More insight can be achieved on the renal function status with a longer follow-up. The other limitation is the lack of metabolic evaluation and stone analysis in our study. Recent studies highlighted the impact of aggressive medical treatment together with metabolic evaluation on post-PCNL stone recurrence and residual stone regrowth (23).

## CONCLUSION

PCNL can be carried out with acceptable complication rates in patients with CKD. Diabetes, peri-operative complications in form of bleeding and recurrent UTI’s are significantly associated with deterioration of renal function. Post-operative complications are significantly associated with negative outcomes and hence one should be cautious to prevent them or manage them aggressively for a successful outcome.
